# Estimation of exponential growth rate and basic reproduction number of the coronavirus disease 2019 (COVID-19) in Africa

**DOI:** 10.1186/s40249-020-00718-y

**Published:** 2020-07-16

**Authors:** Salihu S. Musa, Shi Zhao, Maggie H. Wang, Abdurrazaq G. Habib, Umar T. Mustapha, Daihai He

**Affiliations:** 1 Department of Applied Mathematics, Hong Kong Polytechnic University, Hong Kong, China; 2 Department of Mathematics, Kano University of Science and Technology, Wudil, Nigeria; 3 JC School of Public Health and Primary Care, Chinese University of Hong Kong, Hong Kong, China; 4 Shenzhen Research Institute of Chinese University of Hong Kong, Shenzhen, China; 5 Collage of Health Sciences, Bayero Unuversity, Kano, Nigeria; 6 Department of Mathematics, Federal University Dutse, Dutse, Nigeria

**Keywords:** COVID-19, Reproduction number, Pandemic, Statistical modelling

## Abstract

**Background:**

Since the first case of coronavirus disease 2019 (COVID-19) in Africa was detected on February 14, 2020, the cumulative confirmations reached 15 207 including 831 deaths by April 13, 2020. Africa has been described as one of the most vulnerable region with the COVID-19 infection during the initial phase of the outbreak, due to the fact that Africa is a great commercial partner of China and some other EU and American countries. Which result in large volume of travels by traders to the region more frequently and causing African countries face even bigger health threat during the COVID-19 pandemic. Furthermore, the fact that the control and management of COVID-19 pandemic rely heavily on a country’s health care system, and on average Africa has poor health care system which make it more vulnerable indicating a need for timely intervention to curtail the spread. In this paper, we estimate the exponential growth rate and basic reproduction number (*R*_0_) of COVID-19 in Africa to show the potential of the virus to spread, and reveal the importance of sustaining stringent health measures to control the disease in Africa.

**Methods:**

We analyzed the initial phase of the epidemic of COVID-19 in Africa between 1 March and 13 April 2020, by using the simple exponential growth model. We examined the publicly available materials published by the WHO situation report to show the potential of COVID-19 to spread without sustaining strict health measures. The Poisson likelihood framework is adopted for data fitting and parameter estimation. We modelled the distribution of COVID-19 generation interval (GI) as Gamma distributions with a mean of 4.7 days and standard deviation of 2.9 days estimated from previous work, and compute the basic reproduction number.

**Results:**

We estimated the exponential growth rate as 0.22 per day (95% *CI*: 0.20–0.24), and the basic reproduction number, *R*_0_, as 2.37 (95% *CI*: 2.22–2.51) based on the assumption that the exponential growth starting from 1 March 2020. With an *R*_0_ at 2.37, we quantified the instantaneous transmissibility of the outbreak by the time-varying effective reproductive number to show the potential of COVID-19 to spread across African region.

**Conclusions:**

The initial growth of COVID-19 cases in Africa was rapid and showed large variations across countries. Our estimates should be useful in preparedness planning against further spread of the COVID-19 epidemic in Africa.

## Introduction

Since early 2020, an outbreak of coronavirus disease 2019 (COVID-19), now a pandemic [1–3], caused by the severe acute respiratory syndrome coronavirus 2 (SARS-CoV-2) [1] has hit the world severely. Many countries are facing a rapid increasing trend of confirmed cases. The case-fatality-rate varies wildly from country to country. As of 13 April 2020, about 2 million people have been infected with COVID-19 with over 117 000 death globally (out of which about 90% of the death cases were from United State and Europe) [1, 4].

Africa reported its first case of COVID-19 in Egypt on 14 February 2020 [3, 4]. As of 13 April 2020, a total of 15 207 infected cases were reported throughout Africa, with 831 deaths, giving an overall case fatality rate (CFR) of 5.47%, among them only 3 cases reported in February 2020 [4].

The African region has been described as one of the most vulnerable with the COVID-19 infection [3] in the initial phase, due to the fact that Africa is important commercial partner of China and as a result, large volume of business people travel to the region. Since the epicenter is now in Europe and Ameria, due to the close tie between Africa and countries, African countries face even bigger threat.

Several control measures have currently been taken by most of the African countries to prevent/reduce the spread of COVID-19, especially against case importation from the COVID-19 epicenters. Some of the measures includes travel ban to and from the most COVID-19 hit countries, school closures, temporary ban of gatherings [3, 5, 6]. Nevertheless, the ability to curtail or reduce and control the local transmission after case importation depends largely on how African governments are seriously sustaining the current recommended measures. Due to the fragile health care system, insufficient health workers, lack of water, and sanitizers for maintaining hygiene in the region. African countries need to find their optimal strategies to stop the spread of COVID-19 in its region.

Numerous epidemiological studies have been conducted to understand the transmission dynamics of COVID-19, which is quantified in two key parameters, the basic reproduction number (the expected number of secondary cases that may be caused by a typical primary case during his/her infectious period in a wholly susceptible population, *R*_0_) and the serial interval (time delay between the symptom onset of a primary case and his/her secondary case, SI). High reproductive number and short serial interval imply rapid growth. In the initial phase, the epidemic (number of new cases over time) typically exhibited exponential growth. The basic reproduction number is a function of the exponential growth rate and the serial interval. Studies conducted on the basic reproduction number, exponential growth rate and serial interval [7–11], many of which have shown the *R*_0_ ranges from 2.0 to 4.0, and initial under-reporting/under-detection during the early phases of the outbreak in Wuhan, China.

The aim of this study is to estimate the exponential growth rate and the basic reproduction number of the COVID-19 at the early stage of the pandemic in Africa, which should be valuable in informing the official and public in the preparedness against COVID-19 spread, forecasting the trend, and highlighting the importance of sustaining strict measures in order to curtail the spread of the virus.

## Methods

We obtained the daily number of COVID-19 cases time series data in Africa from World Health Organization (WHO) from 1 March to 19 March 2020 (https://www.who.int/emergencies/diseases/novel-coronavirus-2019/situation-reports/) [4]. Although there were three confirmed cases throughout Africa from 14 February to 29 February 2020, we did not include them as there were no additional case reported until 1 March 2020. In this work, we considered the situation from 1 March 2020 as the number of cases and death started a steady increasing trend.

Referring to recent studies [7, 8], we model the epidemic curve by employing the exponential growth proposed by Ma et al. (2020) [8]. The Poisson likelihood framework is adopted for data fitting and parameter estimation. The intrinsic growth rate (*γ*) was estimated, and the basic reproduction number *R*_0_ computed via *R*_0_ = 1/*G*(−*γ*) with 100% susceptibility for COVID-19 presumed [12]. The function *G*(∙) represents the Laplace transform and the moment generating function of the probability distribution, for the generation interval (GI, the time between the timing of infections of two successive cases) of the COVID-19 [7, 8, 12]. Note that since GI is not observable, we follow conventional approach to use serial interval (timing between symptom onset of two successive cases) as a proxy of GI. Since the transmission chain of COVID-19 in Africa is yet to be fully uncovered, we adopted the SI estimated in Zhao et al. [13]. We modelled the distribution of COVID-19 GI as Gamma distributions with a mean of 4.7 days and standard deviation (SD) of 2.9 days previously estimated by Zhao et al. [11].

Additionally, we quantified the instantaneous transmissibility of the outbreak by the time-varying effective reproductive number. We adopted previous study by Zhao et al. [13] to estimate the effective reproductive number following the SI technic. By employing the renewal equation methods as described by [13], we showed the effective reproductive number of some African countries with reported data for at least 20 days after exceeding 20 cumulative cases in order to show the potential of COVID-19 to spread across the region.

All case reporting data for each country (dates and cumulative numbers of cases) were analyzed and presented graphically using the **R** statistical software (version 3.6.3, released February 2020, available at https://www.r-project.org/).

## Results and discussion

**Fig. 1:**
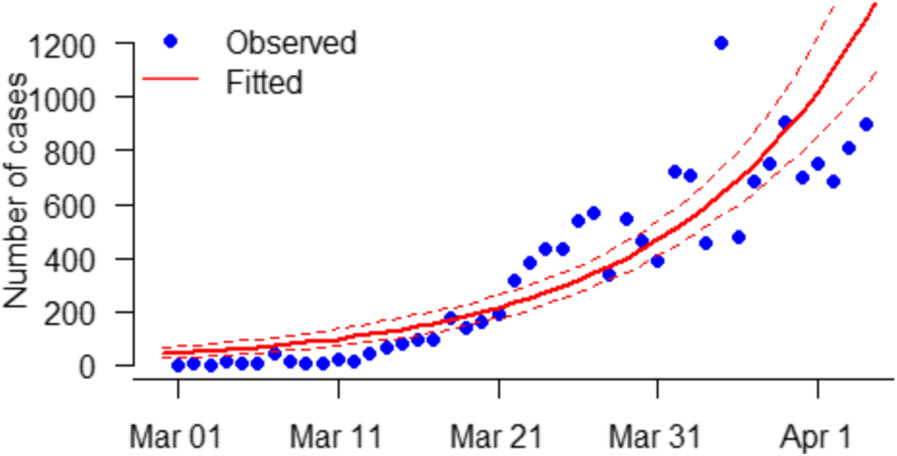
The observed (dots) and fitted (curves) daily number of COVID-19 cases time series in Africa. The blue dots are observations, and the curves are fitting results. The red bold curve represents the mean fitting result, and the red dashed curves are the 95% confidence intervals. Multiple R-squared is 0.76

The exponential growth fitting results are depicted in Fig. 1. The fitting results matched the observed daily number of cases, which implies that the early outbreak data in Africa were largely following the exponential growth rate estimated at 0.22 per day (95% *CI*: 0.20–0.24), which is slightly larger than previous estimates [7, 9, 14]. Our analysis and estimation of *R*_0_ rely on the accuracy of the SI of COVID-19 estimated previously based on cases from Hong Kong, China [11]. We estimated the basic reproduction number *R*_0_ to be 2.37 (95% *CI*: 2.22–2.51), which is also depending on the estimates of the SI during the early epidemics. Our basic reproduction numb *R*_0_ estimates is significantly larger than 1 and broadly consistent with recent studies [7, 14–18]. We suggested that the current COVID-19 outbreaks in Africa could increase rapidly if the measures were not strictly sustained, which includes temporary bans of international travels, avoiding large gatherings, practicing social distancing and so on.

We reported that the mean *R*_0_ of COVID-19 in Africa is likely to be 2.37 which could vary from 2.22 to 2.51 and is consistent with the previous estimates [7, 15, 19, 20]. Denote the infection attack rate (final size of infected) as *z*, then *z* = 1-exp(−*R*_0_*z*), one may solve *z* given *R*_0_ [21] to show this equation is true when the homogeneous mixing assumption holds. Brauer [22] showed that the actual infection attack rate could be low due to public behavior change and heterogeneity in mixing, for instance in the Ebola outbreak, the actual infection attack rate was much smaller than the theoretical estimates in ideal situation. With an *R*_0_ at 2.37, the theoretical infection attack rate will be as large as 87%, namely 87% of Africa will be infected. However, we need to point out that the classical final size overestimated the infection attack rate in the influenza pandemic 1918 and influenza pandemic 2009. These two influenza pandemics had *R*_0_ around 2 and 1.5, respectively. The observed infection attack rate was round 20% in England and Wales, and 10% in Hong Kong, China. Theoretically, the infection attack rate could be 80 and 58%, respectively. The case-fatality-rate (CFR) of 1918 was around 2% [23], and thus the situation of panic and governmental action was similar to COVID-19 pandemic. While the CFR of 2009 influenza pandemic was relatively low, there was virtually little-scale or insufficient control. For these two examples, the expected infection attack rate could be close to the 1918, namely 25%. Thus, there is a need to be prepared and strict action needs to be taken.

**Fig. 2:**
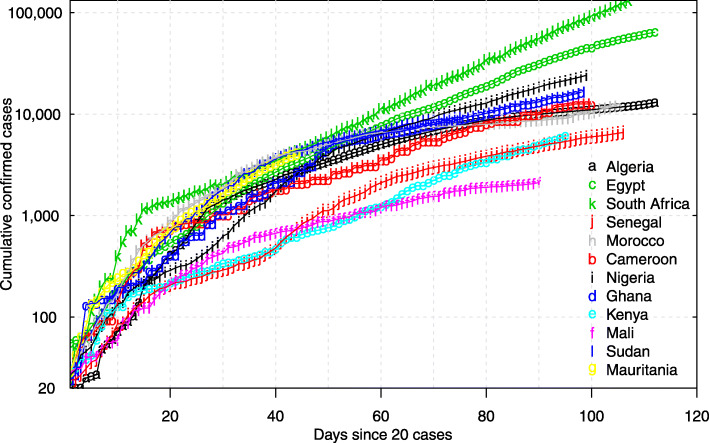
The reported cumulative death confirmations of COVID-19 in top 12 African countries. The horizontal axis is the days since the cumulative exceeded 20 in a country

**Fig. 3:**
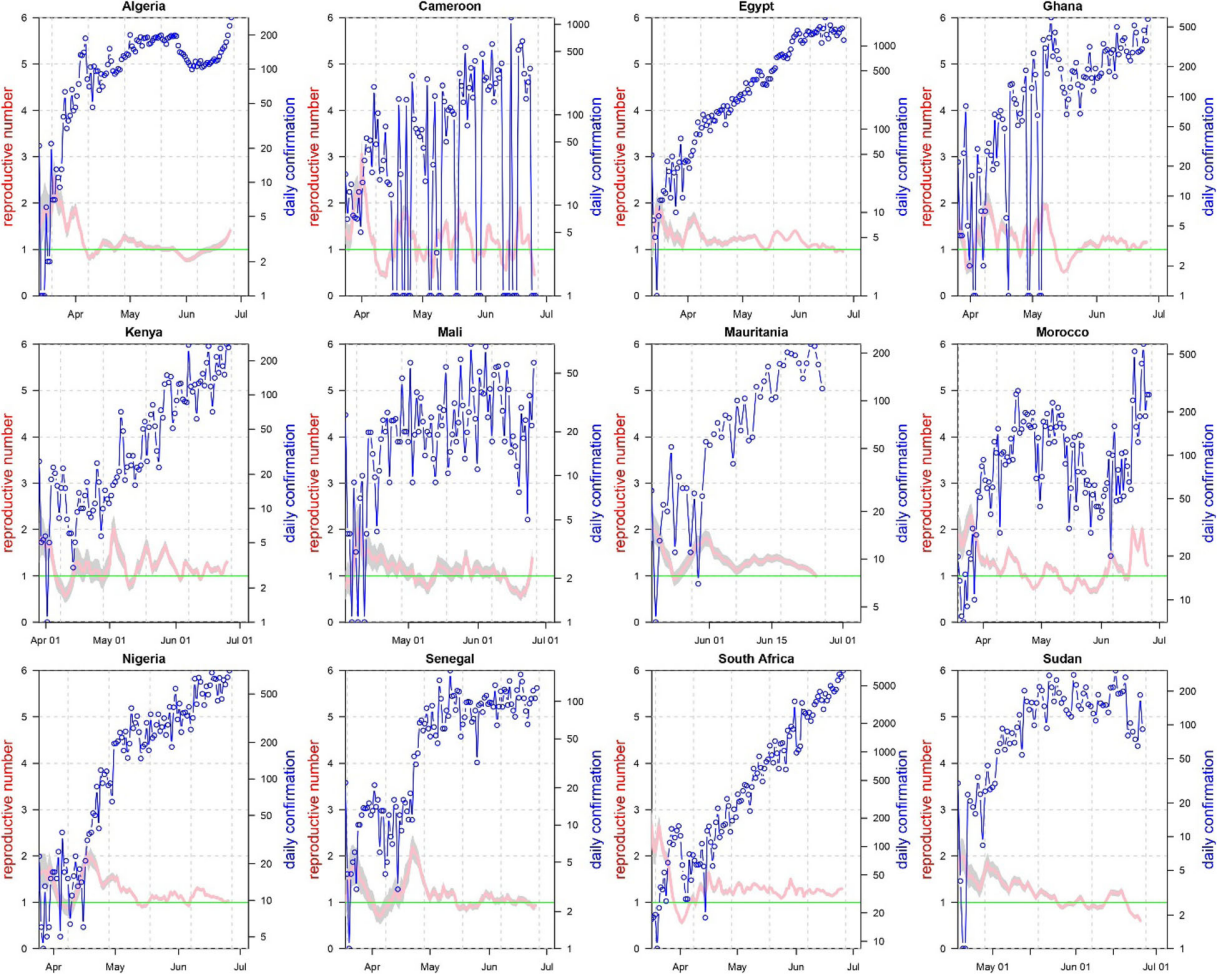
The reported cases (blue cicle line) and the time-varying reproductive number (the three solid lines are the median and 95% *CI*) for the 12 African countries with top number of COVID-19 deaths. CI: Confidence interval

We examined the publicly available materials published by the WHO (WHO, 2020a) to show the potential of COVID-19 to spread without sustaining strict health measures (Fig. 2). We observed the daily confirmations of the COVID-19 death cases in 12 African countries that reported data for at least 20 days after exceeding 20 cumulative cases. Thus, we fitted the cumulative confirmations of 12 African countries starting from the date when cumulative exceeded 20. In Fig. 3, we showed the changing patterns of time-varying reproductive number (*R*_*t*_) based on the daily confirmations of COVID-19 cases time series in Africa. We used the **R** package '*EpiEstim*' to calculate the instantaneous effective reproductive number of 12 African countries in order to show the potential of COVID-19 to spread across the region [24]. After an initial rapid growth in COVID-19 confirmed cases driven by imported cases in the whole Africa, the reported cases showed steady pattern (with decreased reproductive number) in many Africa countries which could be due to the limited diagonostic testing.

We suggested that the likely factors responsible for the rapid increase in the number of confirmed cases in these countries is presumably linked with proportion of population in Africa doing international trade with foreign countries (e.g., China, Germany, Italy and US), also the level of vulnerability of African countries as described by Gilbert et al. [3], i.e., countries with the highest importation risk (such as Egypt, Algeria, and South Africa) followed by countries at moderate risk (such as Nigeria, Ethiopia, Sudan, Angola, Tanzania, Ghana, and Kenya) and then others. This further shows that Africa is one of the most vulnerable region for the COVID-19 pandemic, indicating that there is a need of taking more serious control measures based on the recommendation of WHO and other heath related bodies to curtail the spread of COVID-19 in Africa.

## Conclusions

We estimated the exponential growth rate at 0.22 per day. We estimated the mean *R*_0_ of COVID-19 in Africa at 2.37 with a constant testing effort assumed. We highlighted the importance of sustaining strict health measures in order to contain the virus within a shortest possible time.

## Data Availability

All data used are from public domains.

## Abbreviations

*R*_0_: Basic reproduction number
*CI*: Confidence interval
CFR: Case fatality rate
*γ*: Intrinsic growth rate
*G*(∙): Moment generating function of the probability distribution
GI: Generation interval
*SD*: Standard deviation
